# Exposure and response prevention versus risperidone for the treatment of tic disorders: a randomized controlled trial

**DOI:** 10.3389/fpsyt.2024.1360895

**Published:** 2025-03-03

**Authors:** Jolande M. T. M. van de Griendt, Danielle C. Cath, Agnes A. A. C. M. Wertenbroek, Cara W. J. Verdellen, Judith J. G. Rath, Irene G. Klugkist, Sebastiaan F. T. M. de Bruijn, Marc J. P. M. Verbraak

**Affiliations:** ^1^ Behavioural Science Institute, Radboud University Nijmegen, Nijmegen, Netherlands; ^2^ TicXperts, Heteren, Netherlands; ^3^ Geestelijke GezondheidsZorg Drenthe, Poliklinieken, Assen, Netherlands; ^4^ Department of Psychiatry, University Medical Center Groningen (UMCG)/ Rijks Universiteit Groningen (RUG), Groningen, Netherlands; ^5^ Department of Neurology, Ziekenhuis Groep Twente (ZGT), Hengelo, Netherlands; ^6^ Parnassia Group, PsyQ Nijmegen, Nijmegen, Netherlands; ^7^ Department of Neurology, HAGA Hospital, The Hague, Netherlands; ^8^ Department of Methodology and Statistics, Utrecht University, Utrecht, Netherlands; ^9^ Pro Persona Research, Pro Persona, Arnhem, Netherlands

**Keywords:** tics, Tourette’s disorder, risperidone, behavior therapy, exposure and response prevention

## Abstract

**Introduction:**

The aim of this study was to directly compare behavior therapy (exposure & response prevention; ERP) with pharmacotherapy (risperidone) with respect to tic severity and quality of life in patients with Tourette's disorder or tic disorders.

**Method:**

A total of 30 participants were randomly assigned to either ERP (12 weekly 1-hour sessions) or risperidone (flexible dosage of 1-6 mg) with follow-up at 3 and 9 months after end of treatment. Outcome measures included tic severity as measured by the Yale Global Tic Severity Scale, quality of life and side effects. Predefined informative hypotheses were evaluated using Bayes factors (BF), a Bayesian alternative for null hypothesis testing with p-values, that provides a more reliable and powerful method in the case of small samples. A BF larger than one indicates support for the informative hypothesis and the larger the BF, the stronger the support, with a BF between 3 and 10 being considered to provide moderate evidence.

**Results:**

Both ERP and Risperidone were found to be effective with respect to tic severity at end of treatment (BF 5.35). At 9 months follow-up, results remained stable (BF 4.59), with an advantage of ERP over Risperidone at 3 months follow-up (BF 3.92). With respect to quality of life, an effect was found for ERP (BF 3.70 at 3 months follow up; BF 3.08 at 9 months follow-up). Dropout rates were higher in the medication condition, mainly due to significantly more side effects halfway during treatment, fading out towards end of treatment.

**Discussion:**

Behavior therapy and medication are equally viable options in the treatment of tic disorders, with a slight preference for ERP based on follow-up results on tic severity and quality of life, and side effects.

**Clinical trial registration:**

https://onderzoekmetmensen.nl/nl/node/23410/pdf, identifier NL-OMON23410.

## 1 Introduction

Tourette’s disorder (TD) and chronic tic disorders (CTD) are complex neuropsychiatric conditions characterized by tics; brief, sudden, rapid, recurrent and non-rhythmic motor movements or sounds (1). Tic disorders are quite common, with a prevalence of up to 3-4% for CTD and 1% for TD (2). To date, tic treatment consists of either pharmacotherapy or behavior therapy. In the last decade, more emphasis is placed on non-pharmacological treatments of tics (3); present clinical guidelines recommend to start with behavior therapy before medication (4–7). The primary aim of this randomized, single-blinded, controlled study is to directly compare the effects of behavior therapy to medication in the treatment of tics in patients with TD or CTD.

The two main forms of behavior therapy that are advised by the different guidelines are habit reversal training (HRT) and exposure and response prevention (ERP). Both treatments are designed to intervene in the negative reinforcement cycles maintaining tics, where tics are preceded by premonitory urges. Tics result in a short-time relief of these urges, however, the tic performance by and in itself reinforces subsequent ticcing when premonitory urges re-occur. HRT intervenes in this cycle by stimulating an increase in patients’ awareness of the cycle of premonitory urges followed by tics (“awareness training”) and teaching the patient to replace the tics by incompatible responses(“competing response training”) (8). HRT and its extended version Comprehensive Behavioural Intervention for Tics (CBIT) has been proven effective in several RCT’s (9–13), showing percentages of improvement between 18% and 38% and effect sizes between 0.57 and 1.5. At follow up, these percentages improved between 31% and 46%. Another behavioral intervention for tics is ERP (11), which intervenes by controlling all tics simultaneously (“response prevention”), in the meantime exposing patients optimally to their premonitory urges. Thus, exposure therapy specifically aims at interrupting the association between the premonitory urge and the tic. There is support for the hypothesis that, by confronting patients for a prolonged period of time with the sensations (exposure) and stimulating them to resist the tic (response prevention), patients learn to tolerate the unpleasant sensation preceding their tic (14, 15), resulting in a reduction of tic behavior. ERP is demonstrated to be equally effective as HRT, with an effect size of 1.4 and percentages of 33% improvement in tic reduction directly after treatment, and 47% at 3 months follow-up (after an additional HRT treatment in a cross-over design) (11). Both for ERP as well as for HRT/CBIT, different treatment modalities have been developed to optimize and help disseminate the treatment. For example, treatment can take place online (16–22), in groups (23–26), for very young children (27), and served by different professionals [e.g. nurse practitioners (28) or occupational therapists (29)]. Further, research on the duration and frequency of sessions, there are indications that behavior therapy also works in fewer sessions [4 sessions CBIT in 3 months (30)], sessions of shorter duration [1 hour ERP instead of 2 hour (31)] and in intensified, brief programs (32).

The pharmacological agents mostly used in treatment of tics entail either α – adrenergic agents or Dopamine-2 (D-2) blocking medicines. There is substantial evidence that D-2 blocking medicines are specifically effective in reducing tics, by either blocking or modulating D2 dopamine receptors in striatal and prefrontal cortical areas (33). Risperidone belongs to the group of atypical antipsychotics with D2 as well as serotonin blocking properties, as well as -to a lesser extent- α1- α2-and antihistaminergic properties. Several RCT’s on risperidone in TD/CTD have been performed, comparing it with either placebo (34, 35), or active comparators (aripiprazole (36), pimozide (37, 38) and clonidine (39), all describing positive effects of Risperidone on tic severity. Overall, risperidone seems equally effective as active drug comparisons, with percentages of improvement between 21% and 56% and (for very few studies reported) effect sizes between 0.4-0.9. To the best of our knowledge, no follow-up studies have been performed. Three recent systematic reviews (40–42) confirm the effectiveness of risperidone on tic reduction, as well as a meta-analysis, describing risperidone (together with aripiprazole) as the most robust evidence-based treatment option for the treatment of TD/CTD (43). Finally, the AAN Guidelines (5) indicated moderate confidence that risperidone was probably more likely than placebo to reduce tics, based on 2 Class II studies (34, 35). Overall, risperidone can be considered as a drug with a high level of evidence (7), and in the former European Guidelines the most commonly prescribed medication for tics as rated by European experts (33). Although rather effective in reducing tics, risperidone is also associated with a wide range of adverse effects including sedation, weight gain, orthostatic hypotension and extrapyramidal side effects (44). Relative to placebo, risperidone has a higher risk of drug-induced movement disorders, weight gain, and somnolence (5). Many patients are reluctant to take antipsychotics and up to 70 percent of patients discontinue medication regimes within one yea**r** (45).

To conclude, both behavior therapy and pharmacotherapy seem to be effective in tic disorders, but a direct comparison between the two treatments has only been performed in one study to date (46). In this study, pharmacotherapy (i.e. either risperidone, aripiprazole or pimozide) was directly compared to behavior therapy (either HRT or ERP) and to psychoeducation in children with TD and CTD (n=110). This study showed significant tic reductions for both behavior therapy and pharmacotherapy after 8 weekly sessions with a follow-up period up to 3 months, while psycho-education did not show an effect. No specific results for either ERP or HRT, or a specific medication was presented.

Considering the paucity of studies directly comparing specific pharmacotherapy with a specific form of behavior therapy, the primary aim of this randomized, single-blinded, controlled study was to directly compare the effect of ERP to risperidone in the treatment of tics in patients with TD or CTD. The choice for ERP was based on the fact that ERP is widely used as a treatment for tics in the Netherlands. The choice for risperidone was based on the research above showing that it is a drug with a high quality of evidence and at the time the study started the most commonly prescribed medication for tics in Europe (33). Since RCTs of behavior therapy and medication show roughly comparable effects when looking at improvement rates after treatment, we expected risperidone and ERP to be equally effective. Furthermore, we explored whether ERP had fewer and less severe side effects and lower drop-out rates than risperidone and that ERP had more sustainable treatment effects, while the effects of risperidone were expected to dissipate with drug discontinuation.

## 2 Methods

### 2.1 Design

A baseline assessment was performed, in which in- and exclusion criteria were checked and written informed consent was obtained. Randomization was conducted separately for patients under and over the age of 18 (stratification by age; <18 and ≥18), to prevent imbalance in children and adults between the two groups. All primary and secondary outcome measures were administered by blinded assessors at baseline (week 0), halfway during treatment (week 6), at end of treatment (week 12) and at follow-up (week 24 and 52). Finally, dropout rates and reasons for dropout were collected. Dropout was defined as not completing 12 weeks of treatment.

### 2.2 Patients

**Figure 1:**
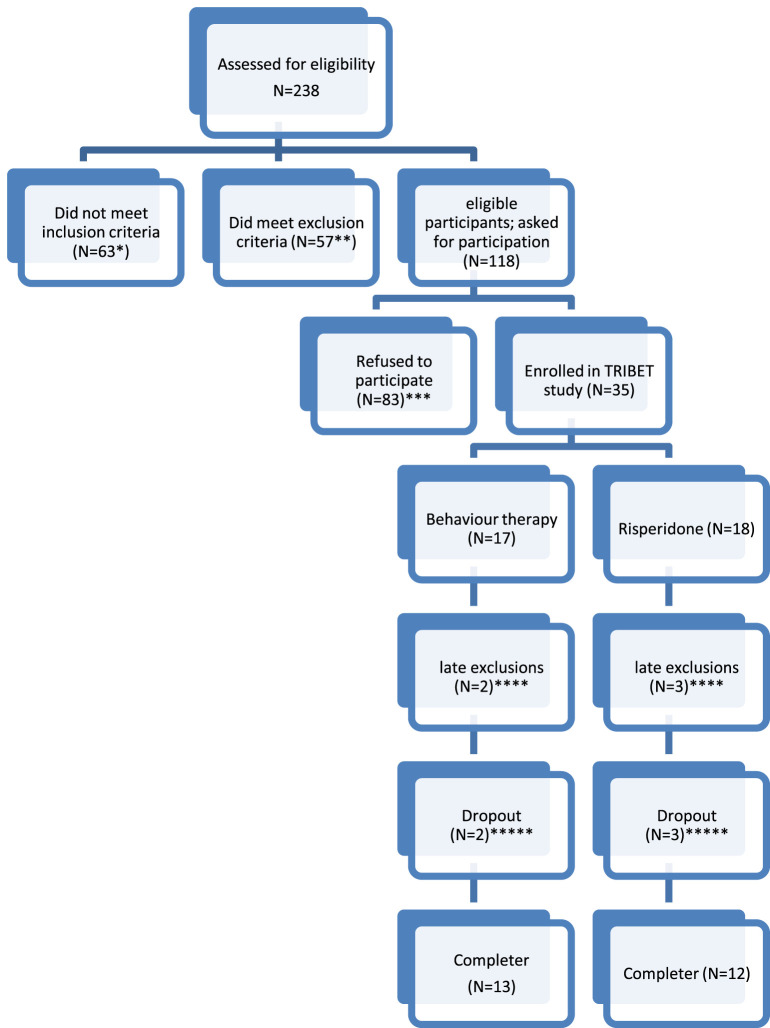
Flowchart of included patients. * No CTD or TD as primary diagnosis (N=25); tic reduction not the primary treatment aim (N=35), YGTSS<13 or in case of only vocal/motor tics YGTSS<9 (N=3). ** Fulfilling a comorbid diagnosis excluding participation in the trial (severe depression (N=4); severe autism (N=3); Low IQ (N=3); prolonged QT-interval (N=2); wish to become pregnant (n=1)); Current use of tic medication (N=44). *** Refusal to be randomized (N=78, of who n=68 refused because they did not want to use medication, n=3 because they did not want to undergo BT and n=7 refused randomization in general); practical problems (N=3); unknown (N=2). **** Withdrawn from study before start: used tic medication (N=1); tic disorder turned out to be a blepharospasm (N=1). Risperidone: refusal of medication after randomisation (N=2); treatment motivation disappeared between randomization and start treatment (N=1). ***** Dropout (defined as not completing the full 12 weeks of treatment): Dropout from behaviour therapy: lack of motivation (N=1); start using medication (n=1). Dropout from risperidone treatment: side effects (N=3).

A total of n=238 patients with TD or CTD were invited to participate. Participants were recruited through the Dutch Tourette Association, general practitioners, psychiatrists, and neurologists from various outpatient services throughout the Netherlands. Patient characteristics including demographics as age, comorbidity, in- and exclusion criteria and duration of disorder were checked at baseline. Although a total of n=118 were eligible for the study, n= 83 patients refused to participate because they had a clear preference for either CBT (82%) or medication (4%) and therefore could not be randomized. From the n=35 that were randomized for the study, 5 participants were withdrawn before start of treatment due to several reasons (see **Figure 1**), so the final sample of participants was n=30. **Figure 1** shows a flowchart of patient inclusion.

Inclusion criteria were a primary diagnosis of TD or CTD, as established with DSM-IV (47) criteria by trained assessors, with at least a moderate severity of tics (YGTSS≥13 or in case of only vocal/motor tics YGTSS≥9). Age ranged from 6 to 65 years of age. Exclusion criteria were severe major depression (with a Beck Depression Inventory (BDI) score in adults (48) of ≥ 30, or a Child Depression Inventory (49) score in children of ≥ 19), autism spectrum disorder (as established in previous treatments, and based on a score on the Autism Questionnaire (AQ) of ≥ 32) (50), current psychotic disorder, addiction, mental deficiency and inability to read/speak Dutch. Comorbidity was assessed at baseline using the Mini International Neuropsychiatric Interview (51) for adults, or the MINI-KID (52) for children. Other exclusion criteria were current use of psychotropic medication to reduce tics, a known prolonged QT interval at ECG, and a pregnancy (wish). Patients needed to be free of specific tic medication (antipsychotics) for at least four weeks prior to entering the study. Written informed consent by patients, as well as from their parents in case of children (<16) was necessary to participate in the study. Participation was voluntary and there was no monetary compensation. However, participants were reimbursed for their transportation costs in relation to the assessment appointments.

The study was approved by the Medical Ethics Committee under file number NL27245.098.09 and registered at the Dutch Trial Register under number NTR2337 (https://onderzoekmetmensen.nl/en/trial/23410). Patients were recruited between August 2011 and December 2013.

### 2.3 Outcome measures

The primary outcome measure used was the Total Tic Score of the Yale Global Tic Severity Scale (53) (Dutch version). The YGTSS is a well-established, semi-structured clinician derived rating scale with satisfactory convergent and discriminant validity and interrater agreement. Information on tic severity was acquired for motor and vocal tics separately in five dimensions: number, frequency, intensity, complexity, and interference. These dimensions were summated and the subscale scores were obtained (Total Vocal Score & Total Motor Score), each ranging from 0-25. Subscale scores were summated into the main outcome parameter, the Total Tic Score, ranging from 0-50. A rating of impairment (ranging from 0-50) was scored separately.

Secondary outcome measurements included quality of life measurement and assessment of side effects. Quality of life was measured using the Dutch translation of the Gilles de la Tourette Syndrome–Quality of Life Scale (GTS-QOL) (54), which is a 27-item, patient-reported scale that measures TD-specific quality of life on 4 subscales (psychological, physical, obsessional, and cognitive subscale). It takes into account the complexity of the clinical picture of TD. The English version of the GTS-QOL demonstrated satisfactory scaling assumptions and acceptability, high internal consistency, high reliability and test-retest reliability, and supported validity.

Side effects were measured in both conditions by the Udvalg voor Kliniske Undersøgelser (UKU) Side Effects Rating Scale (55). The UKU is a comprehensive side effect rating scale with well-defined items and scale steps, developed to be used in clinical drug trials and in routine clinical practice. It comprises ratings (0-4) of 48 single items, a global assessment of the influence of the reported side effects on daily performance, and an item on the effects of the adverse events on continuation of the medication. The items are clustered into four sub-groups: Psychological, Neurological, Autonomic and Other side effects. In the medication condition, the UKU was used every visit (to decide about possible increase or decrease of dosage) as well as during assessments, while in the behavior therapy condition, the UKU was measured during assessments only.

All outcome measures were performed by blinded assessors.

### 2.4 Treatment

Stratified by age (<18 years and ≥18), patients were randomly assigned to either behavior therapy or medication. Treatment took place in one of the four participating locations in the Netherlands (Altrecht in Utrecht, Haga Hospital in the Hague, ZGT Hospital in Hengelo and HSK Group in Den Bosch). Both treatments were available on each location. Behavior therapy was given by trained behavior therapists; risperidone was prescribed by neurologists and psychiatrists.

#### 2.4.1 Medication

The medication condition consisted of a flexible dose of risperidone, between 1-6 mg a day (33). Patients started with one capsule of 0,5 mg at bedtime until day 4, at which point the dose was increased to 2 capsules per day. The clinician reviewed the subject’s response to the dose increase in a telephone session around day 7. Follow-up visits were scheduled every 2 weeks in which the dose was increased in 0.5 mg increments based on clinical effect and tolerability, evaluated on a weekly basis up to a maximum of 6 mg per day. In children with weight below 50 kg, starting doses and increments consisted of steps of 0,25 mg. At each visit, side effects were reviewed, with dose adjustments accordingly. No dosage increase was planned after week 6. Dose reductions were permitted at any time to manage potential side effects. Integrity of treatment was guaranteed by the use of treatment protocols, and a compliance check after 6 weeks of treatment by blood draws to measure blood levels of study medication in a random selection of patients (7 out of 15).

#### 2.4.2 Behavior therapy

The ERP condition consisted of 12 weekly 1 hour sessions following a structured manual (56). The first two sessions were mainly aimed at response prevention of all tics; the therapist encouraged the patients to control all their tics for as long as possible. From session 3 onwards, exposure to premonitory urges was optimized, for example by talking about tics and urges and bringing urge-eliciting objects into the session. By suppressing all tics while focusing on the premonitory urges, patients could learn to tolerate these sensory sensations. Integrity of treatment was guaranteed by the use of a structured manual (56), intensive training and supervision of therapists and monitoring of videotaped sessions. Patients were instructed to practice at home as much as possible, and homework was discussed each session.

### 2.5 Statistical analysis

All analyses with respect to demographics and baseline clinical characteristics were performed using IBM SPSS for Windows version 28.0.1.0 (142). As can be inferred from **Figure 1**, only a low number of patients could be included in the study. A large number of eligible patients had a clear preference for BT, and were unwilling to be randomized to medication. To describe the study population at baseline, the group of patients who received risperidone was compared with the group of patients who received ERP using standard descriptive statistics. For all outcome measures, also descriptive statistics were used to get a first impression of the different patient groups and results over time. We did not use standard inferential statistics to obtain potential evidence for treatment effects, due to small samples. Significance values from traditional (frequentist) tests are not reliable and lack power when samples are too small. Instead, we decided to test predefined informative hypotheses using Bayesian model selection based on the Bayes Factor [BF (57)], analyzed with the software BIEMS (58, 59).

The Bayesian approach offers a solution for inferences from small samples, because there are fewer modelling assumptions (i.e. results are not based on asymptotics) and thus provide more trustworthy results (60). In addition, the formulation and evaluation of informative hypotheses instead of standard null hypothesis testing provides more power to detect support for predefined expectations (61).

Each informative hypothesis that we formulated was evaluated against a hypothesis without constraints on the parameters using the BF. Each BF represents to what extent the data support the constraints. A BF>1 indicates that the hypothesis is supported by the data and a BF<1 implies there is no support. The larger the Bayes factor, the stronger the support for the hypothesis being tested. A BF between 1 and 3 is interpreted as anecdotal evidence, a BF between 3 and 10 as moderate evidence, and a BF above 10 as strong evidence (62, 63). In addition to the BFs, posterior model probabilities (PMP) were calculated to provide a mutual comparison of the support for the competing hypotheses. Assuming that each hypothesis under consideration is equally likely before the data are observed, a PMP is an alternative representation of the information in the BFs. To give an illustration, in the case of three competing hypotheses (as we have in our study) resulting in values BF_1,unc_=2, BF_2,unc_=4, BF_3,unc_=14, the corresponding PMPs would be PMP(H1)=0.10 (computed as 2/(2 + 4 + 14)), PMP(H2)=0.20 and PMP(H3)=0.70. This demonstrates that the PMPs of a set of hypotheses add up to one and represent the relative support found for each hypothesis in the set. For a more elaborate introduction into the Bayesian statistics, we refer to several articles (57, 60, 63), as well as for more information on Bayesian evaluation of informative hypotheses (58, 59, 61, 64). Bayesian statistics are becoming more common in evidence-based research and have been found useful in several studies (65–68), For the Bayesian statistics, the data of completers were used. As a control we ran the same analyses on an intention to treat basis (with imputing of data using the “last measure carried forward” method). Since intention to treat showed no substantial differences with completers, only completers are reported. In order to explore for side effects, non-parametric tests (Independent Samples – Mann-Whitney U test) were used on difference scores between baseline and week 6, and baseline and week 12.

### 2.6 The hypotheses

In our study, we defined three informative hypotheses based on research and expert opinions that were tested on three different time points and on two different measurements. The three different time points were described as an immediate effect (week 0-week 12), a follow-up effect (week 12-week 24) and a long term effect (week 12-52), and measured by the YGTSS-Total Tic Score and the GTS-QoL total score.

The first hypothesis was that both methods would be effective, but ERP was more effective than risperidone. This hypothesis was based on the within effect sizes in the ERP study by Verdellen et al. (1.42) which were larger as compared to within effect sizes found in the risperidone study by Dion et al. (0.46) (11, 35). In this study, this hypothesis was operationalized as higher scores on the YGTSS and GTS-QoL at baseline than directly after treatment (*ERP0>ERP12 & risp0>risp12)* and better treatment effects for ERP than for risperidone (*(ERP12-ERP0)>(risp12-risp0)*). For the follow-up and long term effect, this hypothesis was also in line with the European Guidelines, that state that “An advantage of behavioral treatments may be its better long term effects, beyond the duration of the therapy” (33). This was operationalized as further improvement for ERP at follow up and long term (*ERP12>ERP24 & ERP12>ERP52)*, while the effects of medication stay stable (*risp12=risp24 & risp12=risp52)*.

The second hypothesis was an equality hypothesis (46). In this hypothesis, both ERP and risperidone were expected to be effective (*ERP0>ERP12 & risp0>risp12)*, with no differences between them (*(ERP12-ERP0)=(risp12-risp0)*. At follow-up and long term, results were expected to remain stable for both ERP (*ERP12=ERP24 & ERP12=ERP52)* and risperidone (*risp12=risp24 & risp12=risp52*).

The third hypothesis was a null-effect hypothesis, tested to rule out the possibility that neither ERP nor risperidone would have an effect, or at follow-up and long term even had a negative effect (relapse). This was operationalized as (*ERP0=ERP12 & risp0=risp12)*, at follow-up as (*ERP12<ERP24 & risp12<risp24*), and long term (*ERP12<ERP52 & risp12<risp52*). The informative hypotheses can be found in **Table 1**.

**Table 1:** Informative hypotheses evaluated for each of the outcome measures YGTSS (tic severity() and GTS-QoL (quality of life).

Hypotheses		Formulations		
		Immediate effects (week 0-week 12)	Follow-up effects (week 12-week 24)	Long-term effects (week 12-week 52)
**H1**	*Both ERP and risp are effective and ERP is more effective*. Follow-up/long-term: ERP improves further, risp stays stable	*ERP0>ERP12 & risp0>risp12 & (ERP12-ERP0)>(risp12-risp0)*	*ERP12>ERP24 & risp12=risp24*	*ERP12>ERP52 & risp12=risp52*
**H2**	*Both ERP and risp are effective and equally effective*. Follow-up/longterm: results remain stable	*ERP0>ERP12 & risp0>risp12 & (ERP12-ERP0)=(risp12-risp0)*	*ERP12=ERP24 & risp12=risp24*	*ERP12=ERP52 & risp12=risp52*
**H3**	*Both ERP and risp are not effective*. Follow-up/long-term: symptoms will increase again (relapse)	*ERP0=ERP12 & risp0=risp12*	*ERP12<ERP24 & risp12<risp24*	*ERP12<ERP52 & risp12<risp52*

H, hypothesis; ERP, Exposure and Response Prevention; risp, risperidone.

## 3 Results

A total of 30 patients participated in the study, and were randomized to either behavior therapy (n=15) or medication (n=15). **Table 2** describes the baseline characteristics of the patients. No substantial between-group differences were found in any of the listed variables, both for completers as well as for the intention to treat group.

**Table 2:** Patient characteristics at baseline (N=30).

	risperidone (N=15)		ERP (N=15)	
Percentage of children (<18)	20.0%	n=3	26.7%	n=4
Percentage of males	86.7%	n=13	66.7%	n=10
Percentage of patients with a comorbid disorder*	40.0%	n=6	53.3%	n=8
	*Mean*	*SD*	*Mean*	*SD*
Age at baseline	31.13	13.9	32.27	17.9
YGTSS:				
Total motor score	13.53	4.34	12.60	4.21
Total vocal score	6.93	5.61	5.47	4.37
Total tic score	20.47	8.11	18.07	4.70
Impairment	26.00	16.39	30.00	14.14
GTS-QOL	21.86	20.20	18.20	12.80

* ADHD, OCD, anxiety disorders, mild depressive disorders & impulse control disorder.

Of the 30 patients who started at baseline, n= 25 completed treatment (86%; n=12 in the medication condition, n=13 in the BT condition), with no substantial between-group differences in completer status. At visit 6, the mean dosage of Risperidone was 1.89 mg for adults (SD 0.89, range 1-3 mg), and 1.33 mg for children (SD 0.58, range 1-2mg). A random blood draw in 7 out of 15 patients showed a mean concentration of 3.82 µg/l Risperidone (range 0.50-8.7µg/l) and a mean concentration of 7.7 µg/l 9-hydroxyrisperidone (range 2.5-10 µg/l), which is in line with the advised dosage. At week 24 (FU1), n=22 patients (73%) were available for measurements, as well as n=20 patients (67%) at week 52 (FU2). At FU1, 4 of 12 completers (33%) still used medication in a mean dosage of 1.33 mg (SD 0.58; range 1-2 mg), and 2 patients (17%) had stopped using medication. In 6 patients (50%) the medication status was unknown at week 24. At FU 2, 1 of 12 completers (8%) still used medication; 2 patients did not (17%), and for 9 patients, the medication status was unknown (75%). Furthermore, 4 patients from the medication condition followed between 4 and 15 ERP sessions between week 12 and week 24. Evaluation of these optional additional sessions were not part of the study evaluation.

### 3.1 Results on YGTSS and GTS-QOL

**Figure 2:**
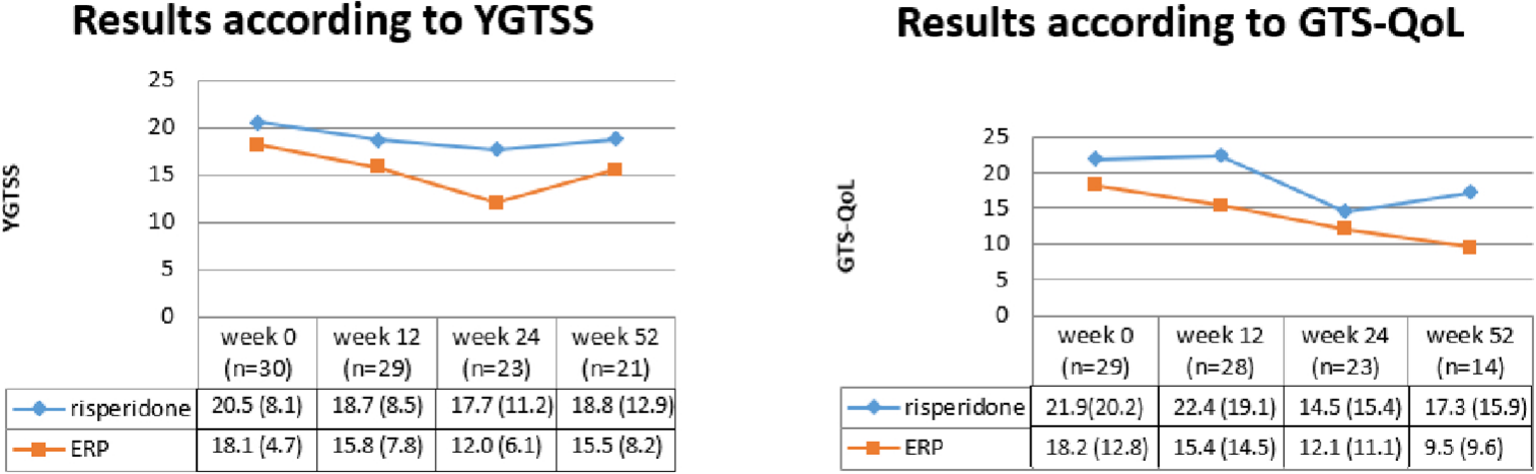
Results according to YGTSS & GTS-QOL [mean (SD)].

In **Figure 2**, the results on YGTSS total tic score (53) and GTS-QOL (54) were shown at the various time points. At the first time point, the results of 29 patients were used for GTS-QOL, since there was a missing GTS-QOL at start of treatment for 1 patient.

Bayes’ factors were computed to investigate which hypothesis best fitted the data. In **Table 3**, the results of the Bayesian analyses can be found.

**Table 3:** Bayesian results on YGTSS and GTS-QOL for immediate effects (Week 0-12), follow-up effects (Week 12-24) and long term effects (Week 12-52), for completers.

		Week 0-12 (N = 25/24^1^)		Week 12-24 (N=21/22)		Week 12-52 (N=20/14)	
		BF_·,unc_	PMP	BF_·,unc_	PMP	BF_·,unc_	PMP
YGTSS	H1	3.87	0.38	3.92*	0.78*	2.52	0.32
	H2	5.35*	0.53*	1.06	0.21	4.59*	0.58*
	H3	0.89	0.09	0.05	0.01	0.79	0.10
GTS-QOL	H1	2.75	0.21	3.70*	0.70*	3.08*	0.82*
	H2	4.60	0.36	1.51	0.29	0.60	0.16
	H3	5.50*	0.43*	0.05	0.01	0.08	0.02

^1^Sample sizes for YGTSS and GTS-Qol, respectively.

*Hypothesis that received most support in the data.

BF**_·_**_,unc_, Bayes Factor of H1/2/3 versus unconstrained hypothesis; PMP, Posterior Model Probability (relative support within the set of H1, H2 and H3); YGTSS, Yale Global Tic Severity Scale; GTS-QoL, Gilles de la Tourette Quality of Life Scale.

Based on the YGTSS, most support was found for hypothesis 2, i.e. equal effectivity of both ERP and risperidone directly after treatment (BF 5.35, PMP 0.53), indicating a moderate evidence for this hypothesis. At week 24, the data seem to support hypothesis 1 (BF 3.92, PMP 0.78), indicating that results on the YGTSS were maintained at follow-up after 24 weeks, with an advantage of further improvement for ERP. At week 52, most support was found for hypothesis 2 (BF 4.59, PMP 0.58), indicating a moderate evidence that both treatments maintain their results in reducing tic severity.

On GTS-QOL, moderate evidence was found for hypothesis 3, indicating no effect of both treatments on quality of life at the end of treatment (BF 5.50, PMP 0.43). However, an improvement at follow up was found for quality of life for patients who followed ERP, while patients in the risperidone group stayed stable, both at week 24 (BF 3.70, PMP 0.70) and week 52 (BF 3.08, PMP 0.82).

### 3.2 Side effects and reasons for dropout

Somatic complaints were measured across both conditions by the UKU (55). After 6 weeks of treatment, significant differences were found for the risperidone condition on tiredness (p=0.013) and weight gain (p=0.005). Patients in the medication condition gained about 3 kg in this period. After 12 weeks, the side effects seemed to have stabilized over the second half of treatment.

Side effects were the main reported reason for dropout in the medication condition (N=3/3), while side effects in the behavior treatment condition did not lead to dropout. Reasons for dropout in the ERP condition (N=2) were motivational issues (N=1) and start of medication during the behavior therapy (N=1).

## 4 Discussion

This study indicates that ERP and risperidone both have a modestly positive effect on tic severity, as well at end of treatment (week 12) as at follow-up (week 24 and 52). This is in line with the conclusion of a previous study that compared both treatments (46), and in line with earlier recommendations of European and American Guidelines (4–6). An advantage for ERP was found at follow-up after 24 weeks on tic severity, and at follow-up after 24 and 52 weeks on quality of life. These data suggest better long-term efficacy of behavior therapy as compared to medication, although Bayes Factors are quite modest. Side effects were higher in the medication condition in the first 6 weeks. During the second half of treatment, side effects seemed to diminish. Side effects included tiredness and weight gain, which is in line with side effects as found in other studies with risperidone (34, 35, 39, 44, 69). It must be noted that numbers of available measurements are too low to draw any firm conclusion on these results. Bayesian statistics showed that quality of life is not affected directly after treatment, nor for ERP, neither for risperidone. The lack of improvement at end of treatment could mean that quality of life might not be directly related to tic severity. Bernard et al. (2009) showed that the correlation between quality of life and tic severity is non-significant when tic severity, as measured by the YGTSS, was mild to moderate. This might suggest that other factors, such as comorbid conditions, might be more influential when it comes to quality of life of individuals suffering from tic disorders. In this study, sample sizes were too small to take comorbidity levels into account. The positive effect on quality of life for ERP suggests that other factors than only tic reduction might be affected by behavioral treatment.

Advantages of this study are that two active treatments that are both recommended in tic treatment, i.e. ERP and risperidone, were directly compared in a randomized controlled study with blinded assessors. However, several limitations need attention. One of the main limitations is the small sample size. Originally, this study aimed at including a total of 80 patients. To solve the issue of the small sample size much smaller than calculated in the initial power calculations, Bayesian statistics were used. Bayesian statistics are a feasible alternative for classical mixed modeling approaches because it works with limited hypothesis testing.

The issue of why we were unable to include more patients in this RCT, needs additional attention. Although many more patients were eligible for the study (118 in total), a high percentage of potential participants refused to take part in the study (83 of these 118 patients (70%)). Of these 83 patients, 82% had a preference for behavior therapy for their tic symptoms and did not want to be randomized because of the 50% chance to be randomized to the medication arm. Both (parents of) children as well as adults had this preference. Even after randomization, 2 patients in the medication condition refused to take medication and were withdrawn from the study. The aversion against medication and preference for behavior therapy as found in this study is in line with earlier reports. For example, Shapiro et al. describe that patients and their parents are reluctant to take medication (45), and two surveys in European TD professionals and Dutch TD patients indicate a clear preference for behavior therapy above medication (6, 70). Moreover, generally RCTs in which two active treatment conditions that are very dissimilar in their nature and characteristics (in our case D2 blocking agents versus behavior therapy) are less suitable with respect to randomization. A basic requirement for randomization is that individuals are “neutral” with respect to the condition to which they are appointed, expecting similar effects and side effects, and similar endeavor to engage in and succeed in the therapy. Especially in medication studies that involve young children, parents as well as clinicians are often reluctant to motivate their children for medication use with direct sedative and long-term potential irreversible side effects (including tardive dyskinesia) when there is a non-medication alternative. This has been the case in this study. Retrospectively, another option could have been to choose a partly randomized patient preference design to enlarge the number of participants (71). Nevertheless, we believe the present cohort to be representative for the general population, although tic symptoms in this study at baseline were rather mild when compared to other studies (mean YGTSS scores at baseline of 20,47 (risperidone) and 18.07 (ERP, compared to baseline scores around 24 and up in other RCT’s (9–13, 23). With mild symptoms, treatment effects are often smaller and more difficult to detect.

In guidelines, behavior therapy is recommended as a first line intervention, and has gained popularity in the last decade. In 2011, 47% of experts considered BT as a first-line intervention, while in 2019 this increased to 63% (in the case of adults) and 79% (in the case of children) (6). The popularity of medication lowered from considering it a first-line intervention by 35% in 2011 to 12% (in case of adults) and 5% (in case of children) in the survey of 2019 (6). However, behavior therapy is often not applied because professionals and patients are unaware of this treatment, and access to it is very limited (6, 72–75). Increasing the availability of therapist by offering behavior therapy training for tics and TD is essential for increasing its accessibility.

Future research could be aimed at replication of the current study including patients with higher tic severity and larger patient numbers. Investigation of the combination of behavior therapy and pharmacotherapy versus one of the treatments alone is relevant too, since to the best of our knowledge this has not been studied so far. Further, patient characteristics that predict differential effects of behavior therapy, medication or a combination especially in patients with persistent tic disorders is warranted, as well as comparing other behavioral treatments for tics (HRT/CBIT) with different kinds of medication. Finally, the reluctance of patients to be randomized in this study warrants further qualitative research into the background of hesitations to use medication. Implementation research is needed of incorporating behavior therapy into routine health care.

Based on this study, behavior therapy and medication should be offered as equally viable options in the treatment of tic disorders, with a slight preference for ERP above medication based on follow-up results and side effects. For routine care, these findings suggest that clear psycho-education about both methods (behavior therapy and medication) is provided, and that patients preferences are in the lead when it comes to choosing treatment.

## Data Availability

The raw data supporting the conclusions of this article will be made available by the authors, without undue reservation.
